# GATA4 blocks squamous epithelial cell gene expression in human esophageal squamous cells

**DOI:** 10.1038/s41598-021-82557-x

**Published:** 2021-02-05

**Authors:** Roman Stavniichuk, Ann DeLaForest, Cayla A. Thompson, James Miller, Rhonda F. Souza, Michele A. Battle

**Affiliations:** 1 Department of Cell Biology, Neurobiology, and Anatomy, Medical College of Wisconsin, Milwaukee, WI USA; 2 Department of Pathology, Medical College of Wisconsin, Milwaukee, WI USA; 3 Department of Medicine, Center for Esophageal Diseases, Baylor University Medical Center and Center for Esophageal Research, Baylor Scott & White Research Institute, Dallas, TX USA

**Keywords:** Oesophageal cancer, Gene expression, Gene regulation, Cell biology, Molecular biology, Gastroenterology

## Abstract

GATA4 promotes columnar epithelial cell fate during gastric development. When ectopically expressed in the developing mouse forestomach, the tissue emerges as columnar-like rather than stratified squamous with gene expression changes that parallel those observed in the pre-malignant squamous to columnar metaplasia known as Barrett’s esophagus (BE). *GATA4* mRNA up-regulation and gene amplification occur in BE and its associated cancer, esophageal adenocarcinoma (EAC), and *GATA4* gene amplification correlates with poor patient outcomes. Here, we explored the effect of ectopic expression of GATA4 in mature human esophageal squamous epithelial cells. We found that GATA4 expression in esophageal squamous epithelial cells compromised squamous cell marker gene expression and up-regulated expression of the canonical columnar cell cytokeratin *KRT8*. We observed GATA4 occupancy in the *p63*, *KRT5*, and *KRT15* promoters, suggesting that GATA4 directly represses expression of squamous epithelial cell marker genes. Finally, we verified GATA4 protein expression in BE and EAC and found that exposure of esophageal squamous epithelial cells to acid and bile, known BE risk factors, induced *GATA4* mRNA expression. We conclude that GATA4 suppresses expression of genes marking the stratified squamous epithelial cell lineage and that this repressive action by GATA4 may have implications in BE and EAC.

## Introduction

Gastroesophageal reflux disease (GERD) is the most prevalent gastrointestinal disorder in the US, with as many as 20% of the population experiencing weekly GERD symptoms^1^. A serious consequence of chronic GERD and its associated inflammatory and cellular stress is the development of Barrett's esophagus (BE), a condition in which a metaplastic columnar epithelium with gastric and intestinal features replaces the normal stratified squamous esophageal epithelium^2–7^. BE, estimated to affect 5.6% of American adults, is most alarming because it is a significant risk factor for esophageal adenocarcinoma (EAC), a deadly cancer with a five-year survival rate of only 19% and that has increased in incidence by more than 400% since 1975^4,6,8,9^. Esophageal cancer is most often diagnosed after symptoms have appeared, marking an advanced disease stage^10^. Given esophageal cancer's aggressive nature and poor prognosis, along with a lack of non-invasive, early screening methods, the mechanisms of BE and EAC development in GERD patients has been the subject of intense scrutiny. Although it is well established that chronic GERD and associated reflux-induced inflammation and cellular damage drive BE development, the precise molecular events of metaplasia are not well understood. Several mechanisms to explain BE have been proposed, but none have yet been proven in human disease^3–5,7^. One possibility is that reflux-induced tissue damage and inflammation cause cellular reprogramming of esophageal stem/progenitor cells^11–14^. Cells residing in submucosal esophageal glands have also been identified as a possible BE origin^15^. Alternatively, stem cells from the stomach or the bone marrow have been implicated in BE^16–19^. Most recently, a population of unique transitional cells at the junction between the stratified squamous epithelium of the esophagus and simple columnar epithelium of the stomach has been identified as a potential source of BE cells^20^. Importantly, these mechanisms are not mutually exclusive, and BE may emerge via multiple routes involving resident esophageal cells and cells from other tissue origins, including the transitional and cardiac zones. Regardless of the origin of metaplastic BE cells, changes in transcription factor expression and activity are paramount in BE pathogenesis. For example, altered transcriptional programs would be necessary to reprogram esophageal stem cells to generate a metaplastic columnar epithelium, direct migration of cells into a damaged esophageal region, or promote survival and maintenance of abnormal, metaplastic columnar cells in the esophageal epithelium. Therefore, transcription factors differentially expressed or differentially active in BE compared with normal tissue are primary candidates for further study. Several have been identified. Columnar epithelial cell-associated transcription factors including CDX2, FOXA2, GATA4, GATA6, HNF4A, HNF1A, and SOX9 are aberrantly expressed in BE and EAC while stratified squamous cell-associated transcription factors including p63 and SOX2 are down-regulated in disease^21–30^. Perhaps not surprisingly, studies have identified networks of these factors at work in esophageal disease^23,27,28^.

Functional studies of candidate BE and EAC associated transcription factors in normal esophageal epithelial cells, esophageal cancer cell lines, and genetically modified mice suggest critical roles for a number of these factors in metaplasia and cancer. For example, when expressed in mouse three-dimensional organotypic esophageal cultures, SOX9 induces columnar-type cytokeratin expression and converts the multilayer structure to a 1–2 cell thick tissue with cuboidal/columnar cell morphology^21^. Squamous lesions develop in the intestine of CDX2 heterozygous mice^31^. Squamous epithelial cells expressing CDX2 are abnormal, resembling multilayer esophagus, a stage proposed as a precursor to full BE metaplasia^29^. Co-expression of CDX2 and Myc in an immortalized esophageal squamous cell line, in conjunction with Notch inhibition, reduces squamous keratin expression and induces columnar keratin and mucin expression^30^. Furthermore, CDX2 expression in transitional cells at the squamocolumnar junction causes BE-like metaplasia^20^. Overexpression of HNF4A and FOXA2 in squamous cells also induces columnar type transcripts and proteins including cytokeratins, villin, trefoil factor, and mucins^13,22^. Exposure of esophageal cancer cells to an acidified media is sufficient to up-regulate GATA6 expression in the cells^23^. On the other hand, loss of squamous type transcription factors causes columnar metaplasia in mice. For example, embryos lacking p63 develop BE-like metaplasia, and hypomorphic SOX2 mutants develop regional columnar metaplasia in the esophagus and forestomach^32,33^.

In this study, we explored the effect of aberrant expression of GATA4 on the epithelial character of mature human esophageal squamous epithelial cells. We focused on GATA4 for several reasons. *GATA4* mRNA is up-regulated in BE and EAC^25,26^. *GATA4* gene amplification occurs in EAC and is associated with poor EAC outcomes^24,26^. Furthermore, we found that GATA4 can promote columnar epithelial cell fate in regions destined to be stratified squamous^34^. When ectopically expressed in the developing mouse forestomach, the tissue emerges as columnar-like rather than stratified squamous, and gene expression changes occurring in the abnormal GATA4-expressing mouse forestomach epithelium parallel those observed in BE^34^. The study presented here extends our studies of mouse squamous versus columnar epithelial development during organogenesis to a human model of the mature esophageal epithelium. Like other studies that have queried the function of BE-associated candidate transcription factors in esophageal cells, we expressed GATA4 in normal human adult esophageal squamous epithelial cells. We tested the hypothesis that ectopic expression of GATA4 in these cells would alter their stratified squamous identity, possibly shifting the cells toward a columnar-like identity. We found that GATA4 expression in these cells caused a loss of squamous epithelial cell marker gene expression and the induction of the canonical columnar cell type cytokeratin *KRT8*. We demonstrated that GATA4 binds to consensus binding sites in the promoters of several squamous epithelial cell defining genes, including the master squamous epithelial cell transcriptional regulator *p63*, implicating GATA4 as a direct repressor of squamous epithelial cell gene expression. As previous studies had identified the expression of *GATA4* mRNA in BE and EAC, we validated GATA4 protein expression in human BE and EAC. Finally, we found exposure of human esophageal squamous epithelial cells to acid and bile, two key reflux components implicated in BE etiology, induced *GATA4* mRNA expression.

## Results

### Ectopic GATA4 expression in human esophageal squamous cells reduces squamous cell marker gene expression

**Figure 1:**
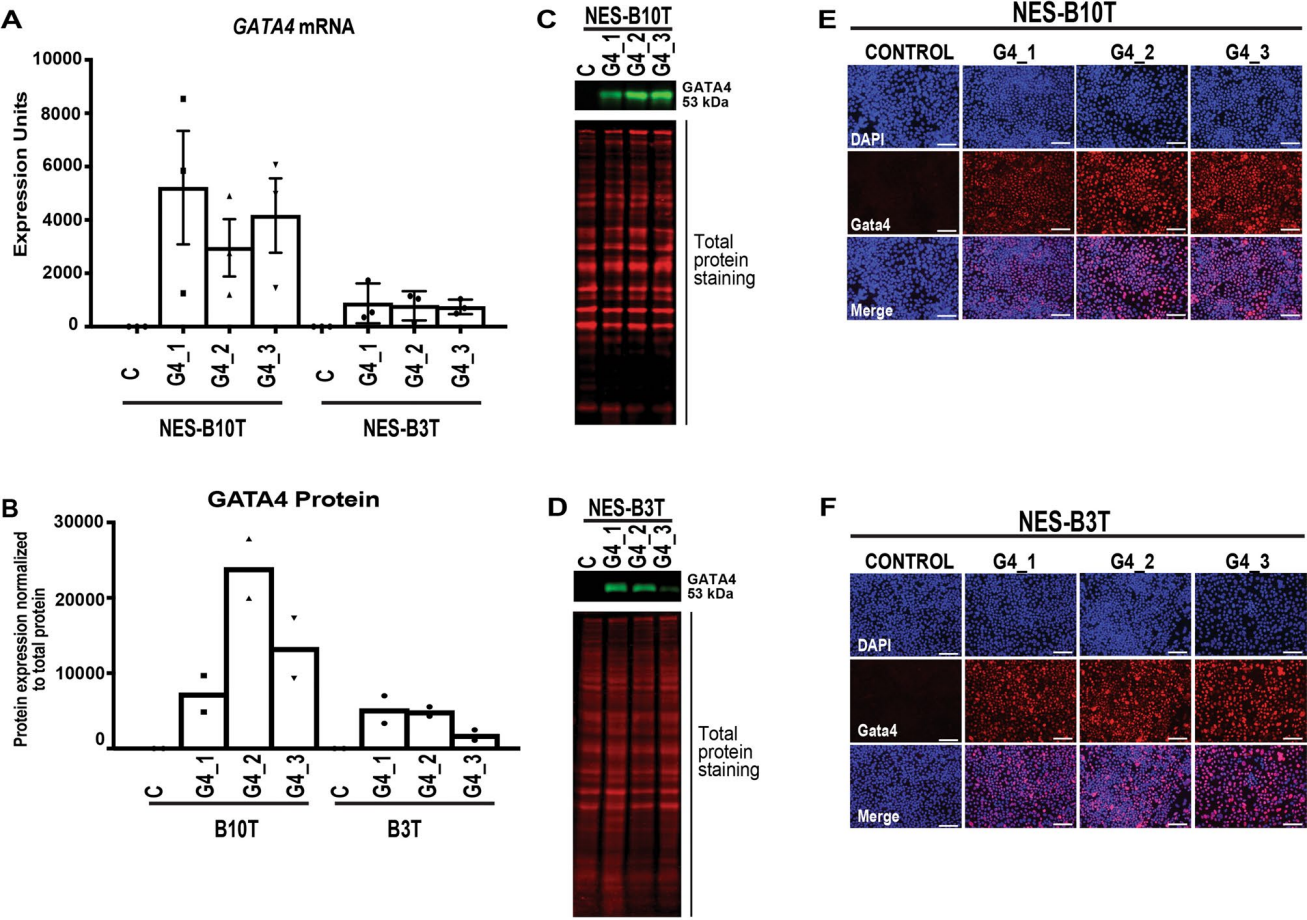
Generation of human esophageal squamous epithelial NES-B3T and NES-B10T cell clones with doxycycline-inducible expression of GATA4 protein. The human esophageal squamous epithelial cell lines NES-B10T and NES-B3T were infected with pInducer20 or pInducer20-GATA4 lentivirus (MOI 3), and clones were isolated after selection with G418. RNA and protein were collected from cells 72 h post doxycycline (1 µg/ml) treatment. (**A**) qRT-PCR demonstrated *GATA4* mRNA induction in three pInducer20-GATA4 clones compared with one control clone per cell line. The data shown represent three independent induction experiments. (**B**–**D**) Quantitative infrared immunoblotting (LI-COR) was used to analyze GATA4 protein expression in nuclear extracts from control and GATA4 expressing B3T and B10T cell clones. Revert Total Protein Stain was used for normalization. Immunoblots were performed using nuclear extracts from two independent doxycycline induction experiments. Representative blots are shown in (**C**) and (**D**). (**E**–**F**) Immunofluorescence staining of GATA4 protein expression (red nuclear staining) in three pInducer20-GATA4 clones compared with one control clone per cell line. DAPI (blue) indicates nuclei. IF detection for GATA4 protein was performed for one induction experiment to determine the distribution of GATA4 expressing cells. Scale bar, 100 µm. Full immunoblots used to generate panels are shown in Supplementary Fig. S1.

We used lentivirus to introduce a doxycycline-regulated GATA4 expression construct or a control construct lacking the *GATA4* coding sequence (vector control) into two human esophageal epithelial squamous cell lines, NES-B3T and NES-B10T^35,36^. Individual independent cell clones with stable integration of the expression construct (n = 3 per cell line) or empty vector construct (n = 1 per cell line) were treated with doxycycline (1 μg/ml) for 72 h before harvesting cells for downstream analyses. We used qRT-PCR to assess *GATA4* mRNA expression in each cell clone (Fig. 1A). *GATA4* mRNA expression was detected in all cell clones containing the inducible expression construct and was undetectable in control cell clones (Fig. 1A). We observed higher levels of *GATA4* mRNA in NES-B10T cells transduced with the GATA4 expression construct compared with NES-B3T cells, with NES-B10T cell clones expressing an average of 5.3-fold more *GATA4* mRNA than NES-B3T cell clones (Fig. 1A).

To examine GATA4 protein expression in control and experimental cell clones, we used immunoblotting (Fig. 1B–D) and immunofluorescent protein staining (Fig. 1E–F). GATA4 protein was undetectable in control cell clones. In contrast, GATA4 protein was present in all six cell clones transduced with the GATA4 expression construct after 72 h of doxycycline treatment. Similar to what was observed between cell lines at the mRNA level, we found that GATA4 protein levels, as measured by immunoblot, were higher in NES-B10T cells transduced with the GATA4 expression construct compared with NES-B3T cells similarly transduced; NES-B10T cells expressed an average of 3.3-fold more GATA4 protein than B3T clones (Fig. 1B).

**Figure 2:**
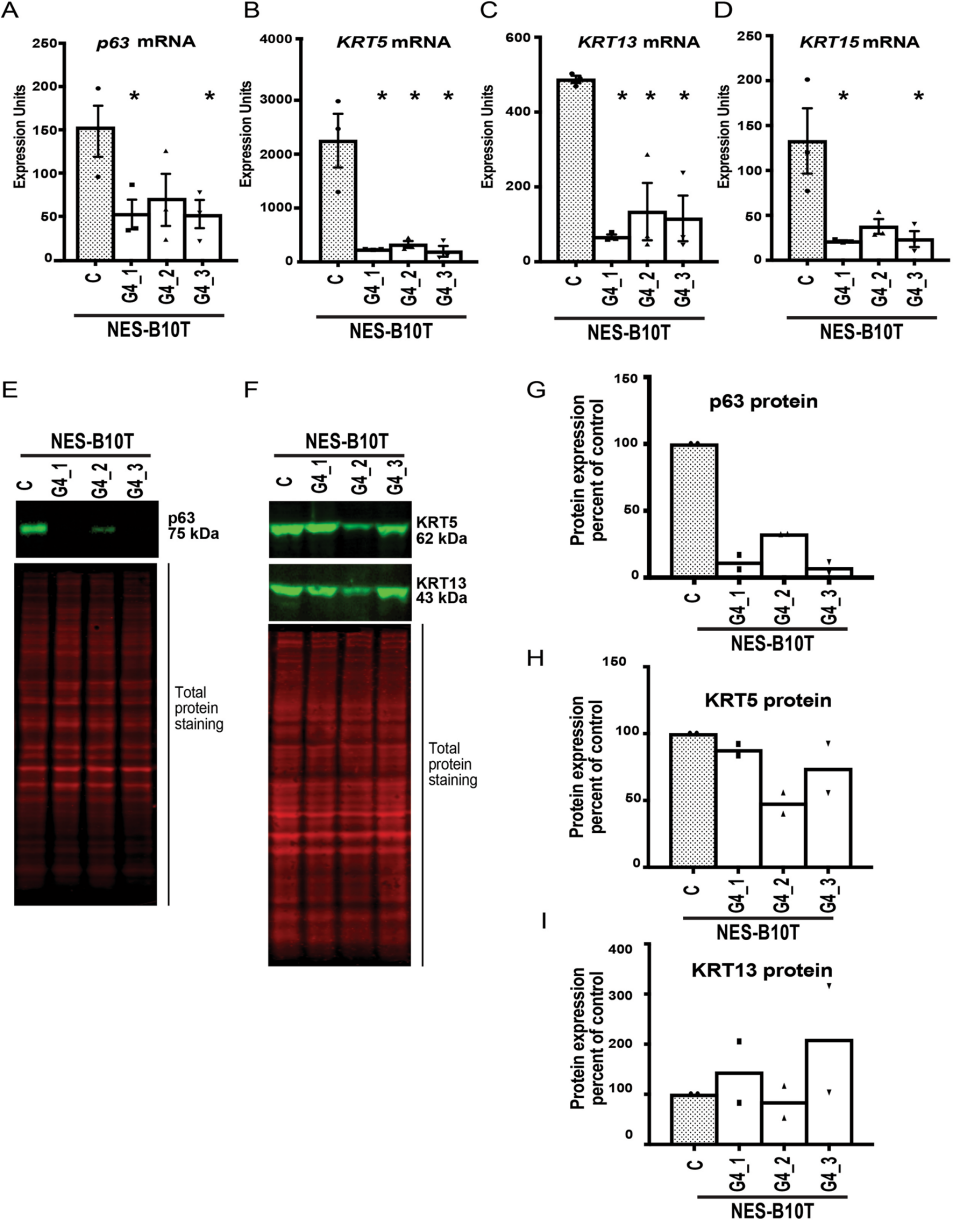
Ectopic expression of GATA4 in human squamous esophageal epithelial NES-B10T cells alters the expression of squamous cell marker genes. Expression of four squamous epithelial cell marker genes *(p63, KRT5, KRT13, KRT15*) were examined by qRT-PCR and/or immunoblot in three pInducer20-GATA4 NES-B10T cell clones (G4_1, G4_2, G4_3) and one pInducer20 NES-B10T control cell clone (C) after 72 h of doxycycline treatment to induce GATA4. (**A**–**D**) qRT-PCR demonstrated reduced steady-state levels of *p63, KRT5, KRT13*, and *KRT15* mRNA in NES-B10T cells expressing GATA4 compared with controls cells lacking GATA4 protein (n = 3 experiments; *P ≤ 0.05). (**E**–**I**) Quantitative infrared immunoblotting (LI-COR) was used to analyze p63, KRT5, and KRT13 protein expression in control and GATA4 expressing NES-B10T cell clones. Revert Total Protein Stain was used for normalization. Immunoblots were performed using protein extracts from two independent doxycycline induction experiments. Representative blots are shown in (**E**) and (**F**). Quantification of blots is shown in panels G-I with the abundance of protein detected in GATA-expressing clones expressed as the percent relative to the non-expressing control cells. Full immunoblots used to generate panels are shown in Supplementary Fig. S1.

To determine the effect of GATA4 on the expression of key markers of a squamous epithelial cell phenotype, we used qRT-PCR and immunoblotting to measure steady-state mRNA and/or protein levels of four canonical squamous epithelial cell marker genes, *p63, KRT5, KRT13,* and *KRT15*, in control and GATA4-expressing NES-B10T and NES-B3T cell clones. We found that expression of GATA4 in NES-B10T cells decreased the abundance of all four mRNAs (Fig. 2A–D), although decreases in *p63* and *KRT15* in clone NES-B10T-G4_2 compared with control cells did not reach statistical significance (p = 0.21 and p = 0.06, respectively). We further examined how changes in mRNA affected the abundance of p63, KRT5, and KRT13 proteins. We observed that NES-B10T clones expressing GATA4 had reduced expression of p63 protein in all clones but that KRT5 and KRT13 levels remained unchanged (Fig. 2E–I).

**Figure 3:**
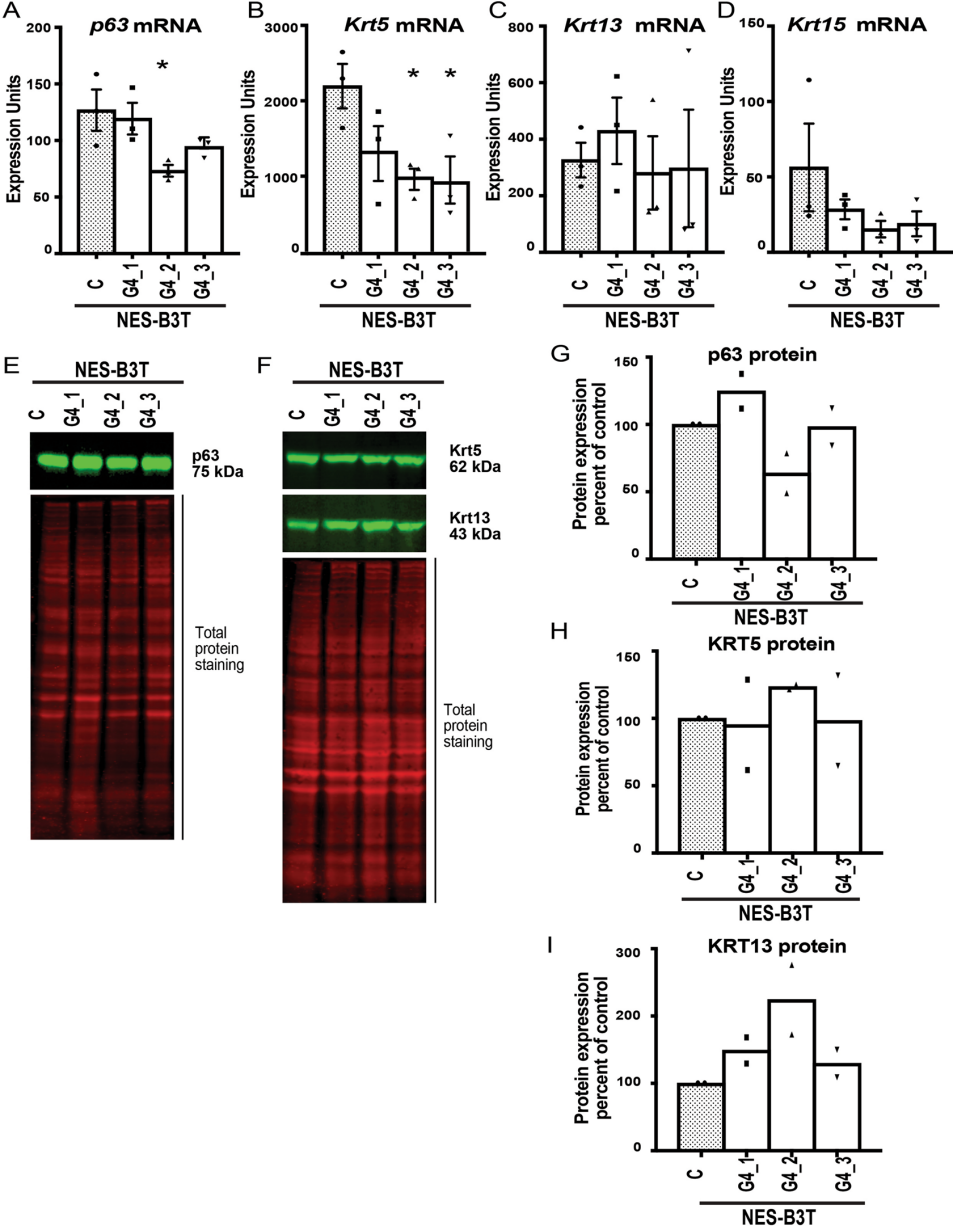
Effects of ectopic expression of GATA4 on the expression of squamous epithelial cell marker genes in human squamous esophageal epithelial NES-B3T cells were less pronounced than those observed in GATA4-expressing NES-B10T cells. Expression of four squamous epithelial cell marker genes *(p63, KRT5, KRT13, KRT15*) were examined by qRT-PCR and/or immunoblot in three pInducer20-GATA4 NES-B3T cell clones (G4_1, G4_2, G4_3) and one pInducer20 NES-B3T control cell clone (C) after 72 h of doxycycline treatment to induce GATA4. (**A**–**D**) qRT-PCR demonstrated a trend for reduction in the steady-state levels of *p63*, *KRT5*, and *KRT15*, but not *KRT13*, in NES-B3T cells expressing GATA4 compared with controls cells lacking GATA4 protein. Statistically significant reductions were observed for only one cell clone, G4_2, for *p63* expression and two clones, G4_2 and G4_3, for *KRT5* expression (n = 3 experiments; *P ≤ 0.05). (**E**–**I**) Quantitative infrared immunoblotting (LI-COR) was used to analyze p63, KRT5, and KRT13 protein expression in control and GATA4 expressing NES-B3T cell clones. Revert Total Protein Stain was used for normalization. Immunoblots were performed using extracts from two independent doxycycline induction experiments. Representative blots are shown in panels (**E**) and (**F**). Quantification of blots is shown in panels (**G**–**I**) with the abundance of protein detected in GATA-expressing clones expressed as the percent relative to the non-expressing control cells. Full immunoblots used to generate panels are shown in Supplementary Fig. S1.

We evaluated the same squamous epithelial cell marker gene expression in NES-B3T cell clones expressing GATA4. Overall, we found that expression of GATA4 in NES-B3T cells generally did not alter the abundance of any of the four marker mRNAs (Fig. 3A–D) or the abundance of p63, KRT5, or KRT13 proteins (Fig. 3E–I). NES-B3T-G4_2 showed a statistically significant change in *p63* and *KRT5* mRNA levels while another clone, NES-B3T-G4_3, showed a statistically significant change in only *KRT5* mRNA levels (Fig. 3A,B). Clone NES-B3T-G4_2 also had a somewhat reduced level of p63 protein compared with control cells (Fig. 3E,G). Another observation emerging from the comparison of NES-B10T and NES-B3T phenotypes was that p63 levels varied between control NES-B3T and NES-B10T cells (Supplementary Fig. S1C). NES-B3T cells expressed approximately tenfold more p63 than NES-B10T cells.

**Figure 4:**
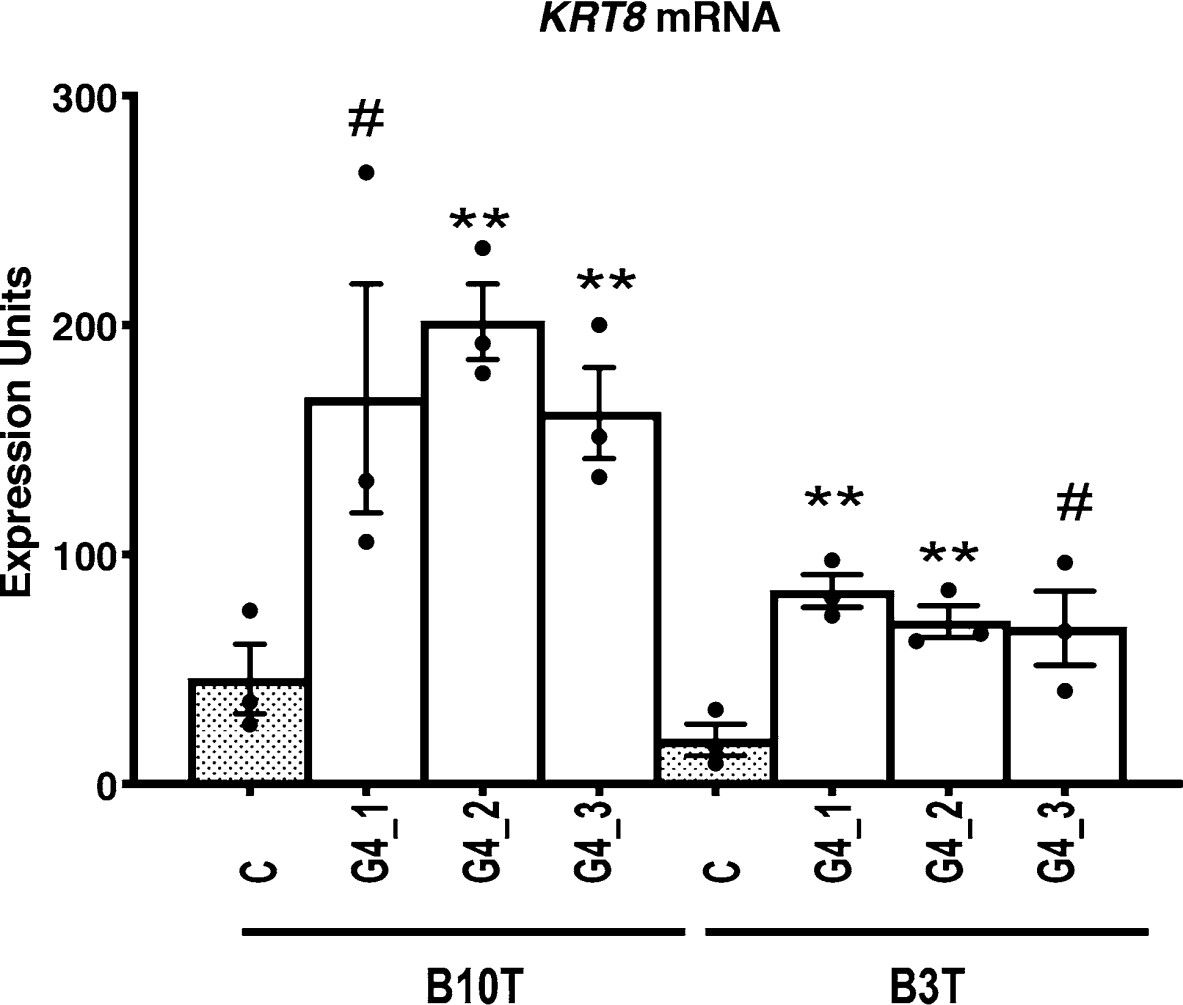
The columnar epithelial cell cytokeratin *KRT8* was induced in human esophageal epithelial NES-B3T and NES-B10T cells expressing GATA4 protein. Expression of the steady-state level of *KRT8* mRNA was examined by qRT-PCR in three pInducer20-GATA4 cell clones (G4_1, G4_2, G4_3) and one pInducer20 control cell clone (C) per cell line after 72 h of doxycycline treatment to induce GATA4. qRT-PCR demonstrated up-regulation of *KRT8* mRNA in NES-B3T and NES-B10T cells expressing GATA4 compared with controls cells lacking GATA4 protein (n = 3 experiments; **P ≤ 0.01; ^#^P = 0.0788, NES-B10T clone G4_1 and P = 0.0503, NES-B3T clone G4_3).

Observing that squamous type keratin gene expression was altered in GATA4 expressing esophageal epithelial cells, we examined mRNA expression of a canonical columnar cytokeratin, *KRT8*, in control and GATA4-expressing NES-B3T and NES-B10T cell clones. We found that control NES-B3T and NES-B10T cell clones expressed low levels of *KRT8* and that GATA4 expression increased *KRT8* expression in all clones by about fourfold (Fig. 4). We surveyed cells for expression of the other columnar markers *CDX2*, *Villin*, and *KRT20* by qRT-PCR and detected no expression of these genes in either control or GATA4-expressing cell clones (data not shown).

### GATA4 binds to promoters of squamous epithelial cell marker genes.

**Figure 5:**
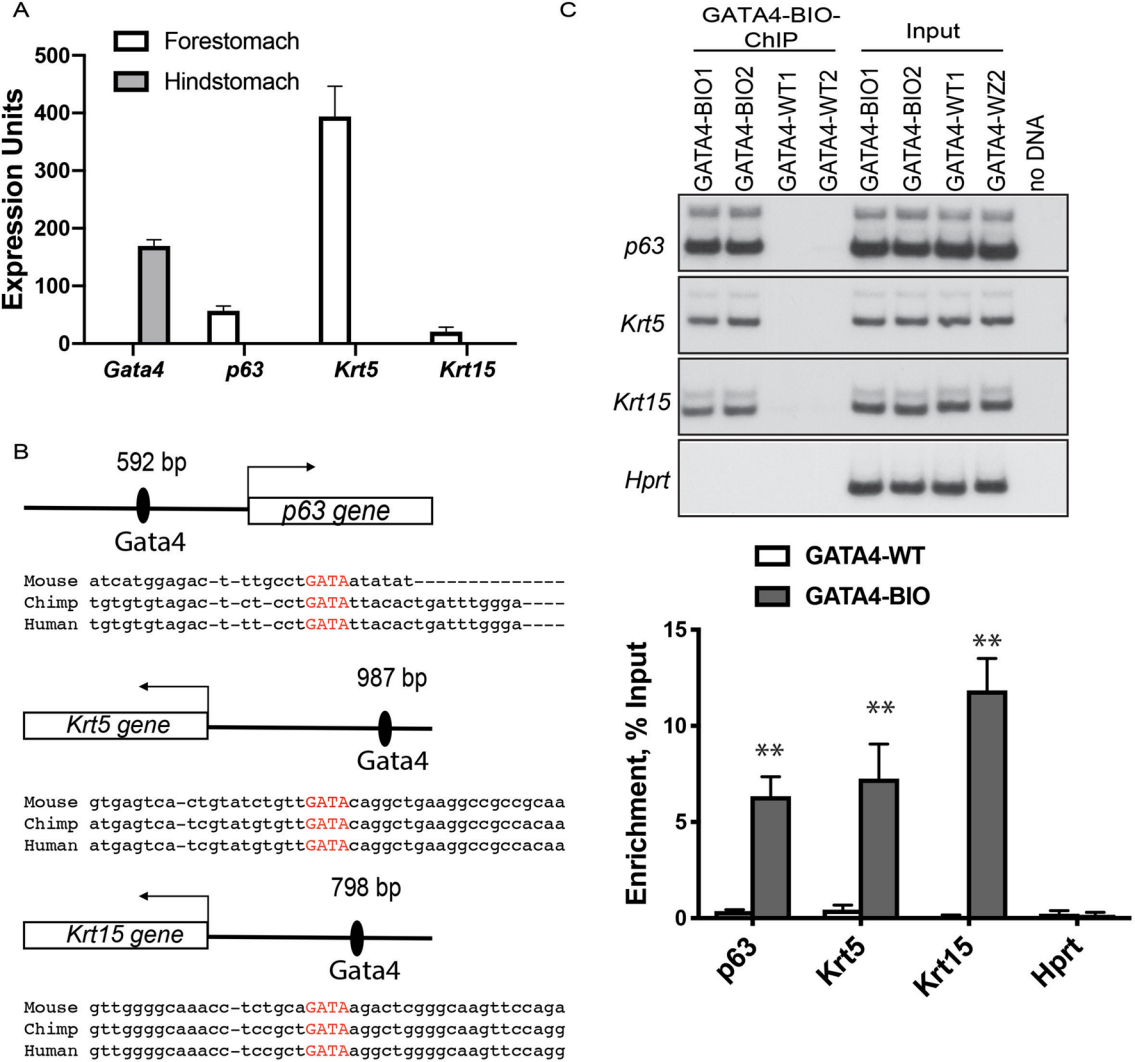
GATA4 occupies the proximal promoters of squamous epithelial cell marker genes suggesting that GATA4 can repress expression of these genes. (**A**) qRT-PCR was used to examine the expression profiles of *Gata4*, *p63*, *Krt5*, and *Krt15* genes in mouse columnar, glandular hindstomach and stratified squamous forestomach epithelial cells. As expected, *Gata4* transcript was detected only in columnar hindstomach epithelium, whereas *p63*, *Krt5*, and *Krt15* transcripts were detected only in the stratified squamous forestomach. (**B**) Diagrammatic representation of GATA consensus binding sites identified as evolutionarily conserved among human, mouse, and chimpanzee in the *p63*, *Krt5*, and *Krt15* genes. (**C**) GATA4 Bio-ChIP–PCR showed enriched amplification of regions containing predicted evolutionarily conserved GATA binding sites in the *p63*, *Krt5*, and *Krt15* genes in cells expressing biotinylated GATA4 compared with control cells lacking biotinylated GATA4. No amplification enrichment was observed for a gene lacking a GATA4 binding site *(Hprt*). Autoradiographic band intensity was measured using a Storm820 Phosphor Imager and ImageQuant software. Representative autoradiographic data for two of four animals per genotype assayed shown. Amplification enrichment per sample was normalized to input (n = 4 GATA4-BIO and 4 GATA4-WT). Error bars show SEM, **P ≤ 0.01. The full autoradiograph used to generate panels is shown in Supplementary Fig. S2.

We next evaluated the capacity for GATA4 to bind to promoters of squamous epithelial cell marker genes. Ideally, we would have performed this experiment in B10T and B3T cells expressing GATA4. However, we were limited by the lack of a well-validated GATA4 antibody for chromatin immunoprecipitation. Therefore, we turned to a mouse model, *Gata4*^*flbio/flbio*^*::ROSA26*^*BirA/BirA*^*,* in which endogenous GATA4 protein is biotinylated (GATA4-BIO) allowing for precipitation of GATA4-bound chromatin complexes by streptavidin-coated beads^37–39^. Because esophageal/forestomach epithelial cells lack GATA4 protein and, therefore, do not allow for direct assessment of GATA4 chromatin binding in stratified squamous epithelial cells, we used hindstomach columnar epithelial cells for this experiment. These cells are adjacent to the stratified squamous forestomach epithelium, express GATA4, and, importantly, lack expression of the squamous epithelial cell genes we propose to be repressed by GATA4—*p63*, *Krt5*, *Krt13*, and *Krt15* (Fig. 5A). We isolated chromatin from columnar hindstomach epithelial cells of *Gata4*^*flbio/flbio*^*::ROSA26*^*BirA/BirA*^ mice (biotinylated GATA4, GATA4-BIO) and *ROSA26*^*BirA/BirA*^ mice (non-biotinylated GATA4, GATA4-WT) (n = 4 animals of each genotype) for bio-ChIP PCR. Using the UCSC genome browser tool with the mouse genome (NCBI37/mm9 build), we visualized gene promoters (defined as 1 KB upstream of the gene's TSS) and identified evolutionarily conserved GATA binding sites within promoters via the genome browser Multiz Alignment tool. We found one GATA site evolutionarily conserved among mouse, human, and chimpanzee in each of the *p63*, *KRT5*, and *KRT15* promoters and no conserved site in the *KRT13* promoter (Fig. 5B). We designed primers flanking the conserved GATA binding sites for PCR from bio-ChIP isolated chromatin. As a negative control, we used primers to assay GATA4 occupancy at a region in the ubiquitously-expressed *Hprt1* gene that lacks GATA4 binding sites and that we have previously validated as a negative GATA4 ChIP control^40–42^. GATA4 enrichment at the putative binding sites in canonical squamous epithelial cell gene promoters in hindstomach chromatin from GATA4-BIO animals compared with that of control GATA-WT animals was measured and expressed as a percentage of input (Fig. 5C). We observed amplification of the binding regions in the promoters of *p63*, *Krt5*, and *Krt15* only in chromatin isolated from GATA4-BIO animals and not from chromatin isolated from control animals (Fig. 5C). As expected, no enrichment was observed for the negative control *Hprt1* (Fig. 5C). These data suggest that GATA4 binds to the *p63*, *KRT5*, and *KRT15* promoters in columnar epithelial cells to repress the expression of these genes.

### GATA4 protein is expressed in human BE and EAC

**Figure 6:**
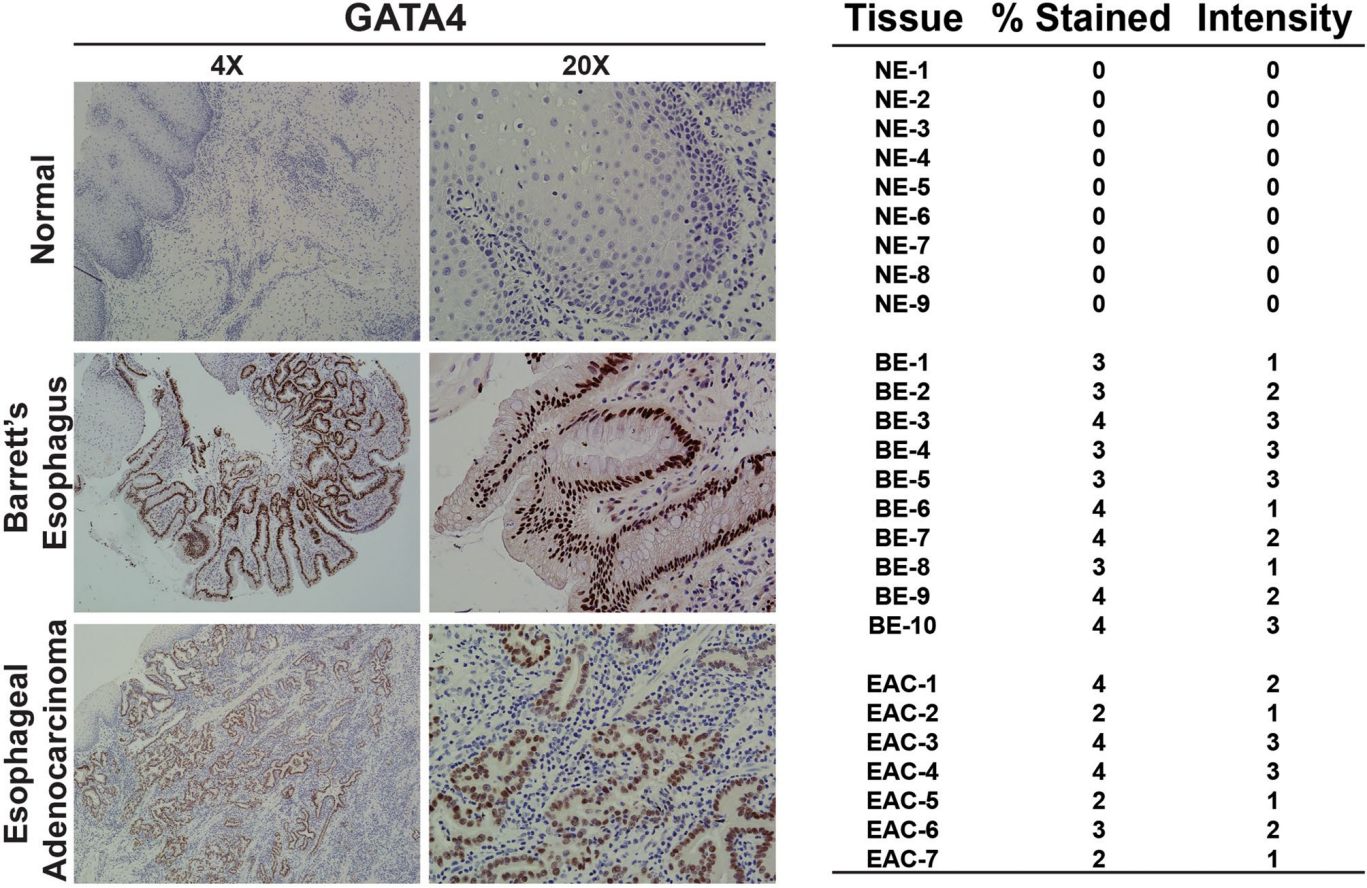
Human Barrett’s esophagus lesions and esophageal adenocarcinoma tumors aberrantly express GATA4. Tissue sections (5 µm) of de-identified human esophageal biopsy tissue without disease (n = 9), with Barrett's esophagus (n = 10), or with esophageal adenocarcinoma (n = 7) were obtained from the MCW Tissue Bank and Department of Pathology. Immunohistochemistry was used to detect GATA4 protein (brown nuclear staining). No GATA4 protein was detected in normal tissue, whereas all BE and EAC tissues analyzed expressed GATA4 protein in diseased epithelial cells. Sections were counterstained with hematoxylin. A board-certified GI pathologist scored GATA4 staining in BE and EAC tissue samples. The scoring rubric used was two-part. The percentage of GATA4 positive cells within the BE or EAC lesion were scored as 0 = 0% positive cells; 1 = 1%-25% positive cells; 2 = 26%-50% positive cells; 3 = 51%-75% positive cells; 4 = 76%-100% positive cells. The GATA4 staining intensity was scored as 0 = no staining; 1 = weak staining; 2 = moderate staining; 3 = strong staining. Scale bar, 100 µm.

*GATA4* mRNA has been reported as abnormally expressed in BE, and *GATA4* gene amplification and expression has been reported in EAC^25,26^. More recently, *GATA4* gene amplification has been associated with poor outcomes in EAC^24^. Furthermore, our study of GATA4 in gastric development demonstrated an overlap between genes differentially expressed in GATA4 mutant forestomach epithelium, which had a columnar rather than squamous cell identity, and BE epithelium^34^. Therefore, because previous studies had not demonstrated GATA4 protein in BE or EAC, we obtained biopsy samples from BE (n = 10) and EAC (n = 7) patients and stained them for GATA4 protein. We detected GATA4 protein in metaplastic regions of all BE biopsies (Fig. 6). We further detected GATA4 protein in all EAC biopsies, although the staining intensity varied among and within tumors (Fig. 6). As expected, none of the normal esophageal biopsy samples (n = 9) examined or regions of normal squamous esophageal epithelium within BE or EAC samples expressed GATA4 (Fig. 6).

### Acid and bile induce GATA4 expression in human squamous cells

**Figure 7:**
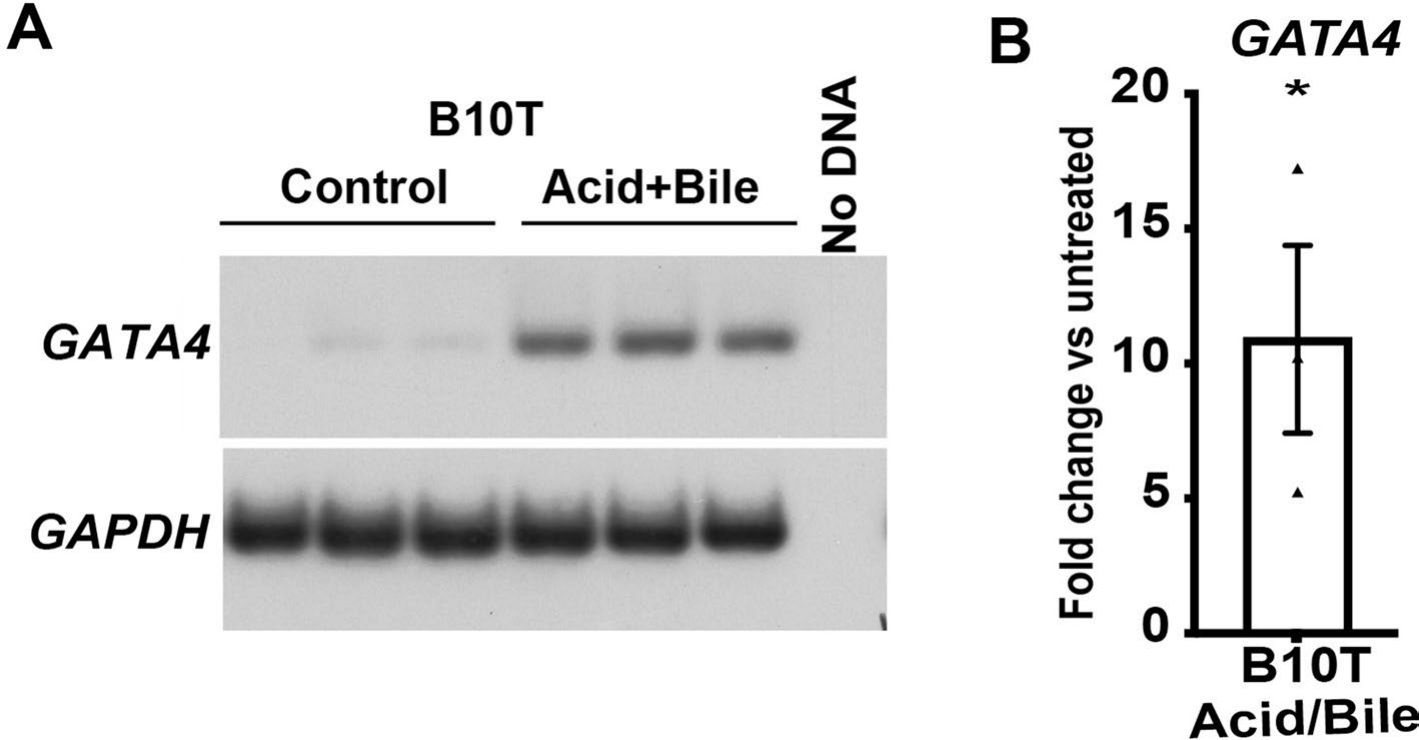
Acid and bile treatment of human esophageal cells in culture induces *GATA4* mRNA expression. The human esophageal squamous epithelial cell line NES-B10T was treated with acidified media (pH 5.5) containing a mixture of bile salts (20:3:15:3:6:1, final concentration 400 mM: glycocholic, taurocholic, glycochenodeoxycholic, taurochenodeoxycholic, glycodeoxycholic, and taurodeoxycholic acids) for 48 h before RNA extraction and semi-quantitative RT-PCR in the presence of [α-^32^P] dATP to detect the steady-state level of *GATA4* mRNA. Amplicons were separated by PAGE and visualized using autoradiography and phosphorimaging. (**A**) Representative image showing *GATA4* mRNA induced by acid and bile treatment of NES-B10T cells. *GAPDH* was used for normalization. (**B**) Phosphorimager quantification of data from three independent experiments demonstrated that *GATA4* mRNA was induced 10.9-fold in acid and bile treated NES-B10T cells (n = 3; *P ≤ 0.05) compared with non-treated control cells. The full autoradiograph used to generate panels is shown in Supplementary Fig. S3.

Finally, we determined the extent to which acid and bile, two key GERD refluxate components implicated in BE, induced *GATA4* mRNA expression. Previous studies have shown that treating human stratified squamous esophageal epithelial cells with acid and bile induces expression of CDX2, an intestinal transcription factor associated with BE^43^. Moreover, *GATA6* is induced in EAC cells treated with acid^23^. We treated control NES-B10T cells with an acidified medium (pH 5.5) containing a mixture of bile salts for 48 h and measured *GATA4* transcript levels by semi-quantitative RT-PCR. We found that acid and bile treatment induced *GATA4* mRNA compared with control untreated cells (Fig. 7).

## Discussion

The study presented here arose from a complementary study we performed to evaluate GATA4 function during gastric development when a common endodermal tissue diverges into two different epithelial cell fates, one being stratified squamous in the esophagus/forestomach region and the other being columnar in the hindstomach region^34^. In that study, we found that GATA4 is essential for columnar epithelial cell development and that when ectopically expressed in developing stratified squamous epithelium, the cells take on a columnar-like identity. Overall, we found that GATA4 promotes wide-scale expression of columnar epithelial cell defining genes and repression of stratified squamous epithelial cell defining genes in the developing stomach to drive normal columnar epithelial cell development. The overlap between BE gene expression profiles and GATA4-direct targets, both positively regulated columnar cell defining genes and negatively regulated squamous cell defining genes, suggests a functional role for GATA4 in disease. These data motivated us to examine how GATA4 ectopic expression in human adult normal esophageal squamous cells would affect their squamous identity. Our data, similar to what we found during gastric development, show that GATA4 depresses squamous epithelial cell defining gene expression when aberrantly expressed in mature human esophageal squamous epithelial cells.

It is clear from our work that individual human esophageal cell lines react uniquely to the ectopic expression of GATA4. We found that NES-B10T cells accommodated a higher level of GATA4 mRNA and protein expression compared with NES-B3T cells. This result is unlikely due simply to positional effects related to lentiviral integration, as three independent cell clones from each line behaved similarly. It more likely reflects inherent differences between these cell lines, and, more globally, may reflect differences between the two individuals from which these lines were derived. Moreover, this result suggests post-transcriptional and post-translational regulation of GATA4 levels given that the expression construct contains only the GATA4 coding sequence and, therefore, lacks endogenous GATA4 regulatory elements. A recent study demonstrates that GATA4 protein is subject to selective autophagic degradation, suggesting that there could be differences in selective autophagy of GATA4 between these two cell lines^44^.

Our data further suggest a dose-dependent effect of GATA4 on human squamous esophageal epithelial cells. In NES-B10T GATA4 cell clones, which expressed about three times more GATA4 protein than NES-B3T GATA4 cell clones, we observed larger changes in expression of the typical stratified squamous cell marker transcripts *p63*, *KRT5*, *KRT13,* and *KRT15*. We further saw a strong downregulation of p63 protein in NES-B10T cells. KRT5 and KRT13 protein levels were not changed in this acute three-day GATA4 induction, and this likely reflects the longer half-life of these cytoskeletal proteins. In general, cytokeratins are long-lived cellular proteins with the average half-life of each spanning around 100 h, which is necessary for their function as cytoskeletal building blocks^45^. In contrast, the transcription factor p63 is short-lived with a half-life of 10–40 h^46^. We predict that longer exposure to GATA4, beyond the acute three-day window examined in this study, would result in downregulation of KRT proteins.

Supporting the idea that NES-B10T and NES-B3T harbor differences that affect their ability to be altered by GATA4 was our finding that baseline p63 levels differ significantly between these cell lines, with NES-B3T cells expressing approximately tenfold more p63 than NES-B10T cells. This variation may be attributable to genetic differences between the donors, or it could reflect differences in physiological or environmental exposures between the individuals from which the cells were derived. One consequence of higher levels of p63 could be resistance to GATA4′s effects on squamous cell reprogramming as we observed that NES-B3T cells did not sustain high GATA4 levels and consequentially showed more subtle effects in stratified squamous cell marker expression compared with NES-B10T cells. Moreover, a higher level of p63 in NES-B3T cells could make these cells more resistant to higher levels of GATA4 protein given that p63 has been implicated in regulating autophagy and GATA4 is a protein targeted for degradation via autophagy^44,47^.

Although we observed alterations in the expression of multiple squamous cell marker genes, we only observed up-regulation of one of the columnar cell marker genes we analyzed, *KRT8*. Interestingly, the effect on *KRT8* was more uniform across the NES-B3T and NES-B10T cell lines, even with the different levels of GATA4. It is possible that longer exposure to GATA4, beyond the acute three-day window examined in this study, would result in further progression toward a columnar cell fate. In fact, a study in which NES-B10T cells were treated with acid and bile for 5 min/day for 30 weeks showed that the expression of markers of columnar epithelium increased progressively over time^12^. Future experiments could test this by inducing GATA4 for more extended periods.

Our data suggest that GATA4 exerts its effects in esophageal epithelial cells by directly binding to GATA consensus sites in the promoters of the *p63*, *KRT5*, and *KRT15* genes to repress expression of these squamous marker transcripts. Although we did not identify an evolutionarily conserved binding site in the *KRT13* gene promoter, this does not rule out the presence of GATA4 regulatory sites beyond the promoter, perhaps in an enhancer. GATA4 is known to activate or repress gene expression. For example, to define jejunal epithelial cells, GATA4 acts on one set of genes positively to promote a jejunal gene expression program and cellular identity while simultaneously acting on another set of genes negatively to repress the ileal gene expression program and its concomitant cellular identity^41,48^. Although we were unable to directly assess GATA4 binding to these promoters in NES-B10T cells because of the lack of a reliable GATA4 ChIP antibody, the evolutionary conservation of these binding sites supports the premise that these sites would be functional in human cells. Based on these data showing GATA4 binding to the *p63*, *KRT5*, and *KRT15* gene promoters in columnar cells and the lack of expression of these genes in columnar cells, we infer that GATA4 could similarly directly repress expression of these genes in human esophageal cells. The differential repressive effects noted on the expression of squamous epithelial cell genes between the two cell lines may reflect differences in expression of essential GATA4 co-factors cooperating with GATA4 to repress squamous gene expression.

Our observation that human esophageal squamous epithelial cells respond to acid and bile by inducing *GATA4* mRNA expression suggests that cells residing within the esophagus in vivo could similarly have the capacity to ectopically induce *GATA4* expression in the presence of reflux, providing a possible link between chronic GERD and GATA4-expressing metaplastic cells. An acid and bile-rich reflux environment could support and stabilize abnormal GATA4-expressing metaplastic cells. Extrapolating from data presented here, we propose a mechanism whereby aberrant GATA4 expression in abnormal esophageal cells, possibly induced by reflux conditions, could support a columnar metaplastic cell identity program by repressing expression of key genes required for stratified squamous cell identity. We acknowledge that the source of BE cells remains controversial and that this study, focused on how aberrant GATA4 acts in squamous esophageal cells, primarily relates to the idea that resident esophageal epithelial cells contribute to BE. Interestingly, esophageal submucosal gland cells and transitional cells, two other purported sources of BE cells, share some qualities with stratified squamous cells including expression of squamous cell markers such as p63 and KRT5, two genes that our study suggest as negatively regulated by GATA4^15,20^. It may be that our studies of GATA4 in esophageal squamous cells will apply to those models of BE. GATA4 expression, however, remains to be investigated in those cell types. As new cell, organoid, and animal models emerge to study BE, particularly those that allow molecular studies of esophageal submucosal gland and transitional cells, it will be essential to examine GATA4.

## Methods

### Tissue immunohistochemistry

Sections (5 µm) of formalin-fixed, paraffin-embedded de-identified human tissue biopsy samples were obtained through the MCW Tissue Bank and Department of Pathology and stained with hematoxylin and eosin. A board-certified GI pathologist verified the presence of Barrett's esophagus or esophageal adenocarcinoma in tissues. Standard immunohistochemical procedures were followed^41,48,49^. Briefly, citric acid antigen retrieval was performed before immunohistochemistry for GATA4 (R&D Systems, AF2606). Staining was visualized using RTU Vectastain Elite ABC reagent and a Metal Enhanced DAB substrate kit. Micrographs were captured using a Nikon Eclipse TE300 fluorescent microscope and SPOT RT3 camera. Images were assembled into figures using Adobe Photoshop and Illustrator. Images from control and experimental samples were processed identically.

### Cell culture

The NES-B3T and NES-B10T telomerase-immortalized human squamous esophageal cell lines were established from biopsy samples of individual patients with Barrett’s esophagus, but not from the diseased region^35,36^. For expansion and maintenance, cells were co-cultured with mitomycin-C inactivated mouse embryonic fibroblast feeder cells in DMEM/F12 (3:1) medium containing hydrocortisone (0.4 mg/ml), EGF (20 ng/ml), transferrin (5 mg/ml), insulin (5 mg/ml), cholera toxin (10 ng/ml), triiodothyronine (20 pM), adenine (31 mg/ml), cosmic calf serum (1%), penicillin/streptomycin (100 U/ml each). Cells were cultured at 37 °C in a humidified 5% CO_2_ incubator. For experiments, cells were cultured on collagen IV coated plates in the same medium.

### Acid and bile treatment of cells

Cells (70% confluency) were treated with an acidified medium (pH 5.5) containing bile salts (20:3:15:3:6:1, final concentration 400 mM: glycocholic, taurocholic, glycochenodeoxycholic, taurochenodeoxycholic, glycodeoxycholic, and taurodeoxycholic acids) for 48 h as previously described^43^. The pH of the medium was adjusted to pH 5.5 immediately before treatment using 1 M hydrochloric acid. After 48 h, cells were flash-frozen in RLT buffer containing β-mercaptoethanol for RNA extraction.

### Lentiviral GATA4 vector transduction and cloning

NES-B3T and NES-B10T cells were infected with pInducer20 or pInducer20-GATA4 lentivirus (MOI 3) prepared by the Medical College of Wisconsin viral core. Plasmids were generously provided by Dr. Stephen Elledge (Harvard Medical School)^44^. Infected cells were maintained in culture medium supplemented with Tet system approved FBS (5%). Cells were selected with G418 (350 mg/ml, B3T; 500 mg/ml, B10T) beginning 24 h after infection. The G418 dose was established as double the concentration required to kill non-infected cells effectively. A limiting dilution cloning method was applied to obtain individual clones, and cells were plated onto collagen IV coated 96-well plates. For experiments, doxycycline (1 μg/ml) was applied to all cell cultures (control and GATA4-transduced) for 72 h. Media was replaced after 48 h to replenish doxycycline. Cells were treated with trypsin to harvest for RNA and protein extraction or fixed with 4% PFA for immunofluorescence experiments.

### Reverse-transcription PCR (RT-PCR)

Total RNA was isolated using the RNeasy Mini Kit. To remove genomic DNA, total RNA was treated with ezDNase. For quantitative reverse-transcription polymerase chain reaction (qRT-PCR), cDNA synthesized using MMLV and random hexamer primers was amplified using TaqMan Gene Expression Mastermix and TaqMan gene expression assays (*GATA4*, HS00171403_m1; *KRT5*, Hs00361185_m1; *KRT8*, Hs01595539_m1; *KRT13*, Hs00357961_g1; *KRT15*, Hs00951967_m1; *KRT20*, Hs00300643_m1; *p63*, Hs00978340_m1; *CDX2*, Hs01078080_m1; *VILLIN*, Hs01031724_m1; *Gata4*, Mm00484689_m1; *p63*, Mm00495793_m1; *Krt5*, Mm01305291_g1; *Krt15*, Mm00492972_m1). Data were normalized to the expression of glyceraldehyde-3-phosphate dehydrogenase (*GAPDH*, 4352665; *Gapdh*, 4351309). For each target, expression units were calculated using the formula [2^(-dCq)^] × 1000^50^. For radioactive semi-quantitative RT-PCR with [α-^32^P] deoxyadenosine triphosphate, cDNA was generated from ezDNase treated RNA using a Superscript VILO cDNA synthesis kit. Primers used were *GATA4* (5′-ttctggggagagtgtaagtggacag-3′, 5′-ctttttgcctcctggacaaaagact-3′) and *GAPDH* (5′-gacagtcagccgcatcttct-3′, 5′-ttaaaagcagccctggtgac-3′). Radioactive amplicons were separated by 4% PAGE, and gels were dried. Expression was detected and quantified using Storm80 Phosphor Imager (Amersham Biosciences).

### Immunocytochemistry

Standard immunocytochemistry methods were used^49^. Briefly, cells were fixed with 4% PFA/1X PBS, permeabilized with 0.5% Triton X-100/1X PBS, blocked with 3% BSA/1X PBS, and incubated with antibodies (GATA4, R&D Systems, AF2606; Alexa-Fluor 594 Donkey anti-goat, Thermo Fisher) in 1% BSA/1X PBS. DAPI was used to visualize nuclei. Images were taken with Nikon Eclipse TE300 fluorescent microscope equipped with SPOT RT3 camera with 20X objective. Images were assembled into figures using Adobe Photoshop and Illustrator. Images from control and experimental samples were processed identically.

### Immunoblot analysis

Standard immunoblot methodology was followed^41,49^. Nuclear extracts were prepared using the NE-PER Nuclear and Cytoplasmic Extraction Reagents and HALT protease inhibitor cocktail. Benzonase (0.5 U/μl) was used to increase protein yield of transcription factors and quality of nuclear extracts. Nuclear extracts (5 μg) or cytoplasmic extracts (20 μg) were separated using Nu-PAGE Bis–Tris 4–12% gradient gels and transferred to an Immobilon-FL polyvinylidene difluoride membrane. Revert Total Protein Stain was used to detect total protein. Blots were blocked with Odyssey blocking buffer for 1 h at room temperature. Antibody (GATA4 D3A3M, Cell Signaling Technology, #36966; KRT5, Covance, #PRB-160P; KRT13, Abcam, #92551; p63 D2K8X, Cell Signaling Technology, #13109) was added to blocking buffer containing 0.1% Tween and incubated with shaking at at 4 °C overnight. Membranes were washed and exposed to a secondary antibody (IRDye 800CW, donkey anti-rabbit, LICOR) for 1 h at room temperature. Blots were visualized using an Odyssey Infrared Imaging System (LI-COR) running Image Studio 5.2 acquisition and analysis software (https://www.licor.com/bio/image-studio). REVERT total protein stain was used for normalization. If required, membranes were stripped with 2% SDS, 62.5 mM Tris buffer, pH 6.8, containing 0.8% β-mercaptoethanol at 50 °C for 45 min. Stripped membranes were washed extensively under running tap water for 2 h. Total protein was re-quantified using REVERT total protein stain, and proteins of interest were detected as described above.

### Animals

*Gata4*^*flbio/flbio*^(*Gata4*^*tm3.1Wtp*^) and *ROSA26*^*BirA*^*(Gt*(ROSA)*26Sor*^*Tm1[birA]Mejr*^*)* mouse lines were used to generate *Gata4*^*flbio/flbio*^*::ROSA26*^*BirA/BirA*^ (GATA4-BIO, biotinylated GATA4) and *ROSA26*^*BirA/BirA*^ (GATA4-WT, non-biotinylated GATA4) mice for biotin-mediated chromatin immunoprecipitation PCR (bio-ChIP-PCR)^37–39,41^. Primers for PCR genotyping of ear punch DNA were *Gata4* Exon 7F/R, 5′-cagtgctgtctgctctgaagctgt-3′, 5′-ccaaggtgggcttctctgtaagaac-3′; BirAJax 14/15 5′-ttcagacactgcgtgact-3′, 5′-ggctccaatgactatttgc-3′; and BirAJax 16/17 5′-gtgtaactgtggacagaggag-3′, 5′-gaacttgatgtgtagaccagg-3′^41^. The Institutional Animal Care and Use Committee of the Medical College of Wisconsin approved all animal procedures. All experiments were performed in accordance with relevant guidelines and regulations.

### Bio-ChIP-PCR

Hindstomach epithelial cells from 2 to 4-month-old GATA4-BIO or GATA4-WT mice (n = 4 animals per genotype) were obtained as previously described with minor changes^41^. Briefly, mouse hindstomach epithelia were separated into single cells by vortexing dissected, washed hindstomach tissue for 30 min at 4 °C in a balanced sodium salt solution (BSS) buffer containing EDTA (1.5 mmol/l KCl, 96 mmol/l NaCl, 27 mmol/l Na_3_C_6_H_5_O_7_, 8 mmol/l KH_2_PO_4_, 5.6 mmol/l Na_2_HPO_4_, and 15 mmol/l EDTA, 200 μmol/l phenylmethylsulfonyl fluoride). Following removal of mesenchymal and muscle tissues, epithelial cell isolates were pelleted by centrifuging at 2000 rpm for 8 min at 4 °C. Cells were washed twice with 1X PBS containing 200 μmol/l phenylmethylsulfonyl fluoride. Bio-ChIP was performed per previously published protocols^38,39,41^. Briefly, cells were fixed with 1% formaldehyde for 10 min, quenched with glycine (final concentration 125 mM), and flash frozen. Cells were lysed and sonicated in 2% SDS buffer using Bioruptor Pico for 6 total cycles, 30 s sonication/30 s rest for each cycle. GATA4 bound chromatin was isolated using magnetic streptavidin beads. After washes and proteinase K/ribonuclease A treatment, chromatin was purified by phenol/chloroform extraction and ethanol precipitation. Chromatin shearing size was determined by Agilent 2200 TapeStation with high sensitivity screen tape. Concentration was measured by Qubit dsDNA HS Assay Kit. Using the UCSC genome browser tool with the mouse genome (NCBI37/mm9 build), we visualized gene promoters (defined as 1 KB upstream of the gene's TSS) and identified evolutionarily conserved GATA binding sites within this region via the genome browser Multiz Alignment tool. GATA4 occupancy of evolutionary conserved GATA binding sites in the upstream regulator regions the *p63*, *Krt5* and *Krt15* genes was validated by radioactive PCR with [α-^32^P] deoxyadenosine triphosphate and gene-specific primers (*p63*, 5′-gcctacattcagaaaggaaacaaattc-3′, 5′-gcctgtcaatggggaaaaataaagt-3′; *Krt5*, 5′-tattccaccagggaagacgtgagt-3′, 5′-tttgggccagagatagaggaaacac-3′; *Krt15,* 5′-aagtctgattgatcaccctgtcacc-3′, 5′-tttctggaacttgcccgagtcttat-3′). As a negative control, GATA4 enrichment was assessed using primers for an exon in the *Hprt* gene (5′-agcgcaagttgaatgtgc-3′, 5′-agcgacaatgtaccagag-3′), which we had previously validated as spanning a region without GATA4 binding^40–42^. Amplicons were separated using 4% PAGE, and gels were dried. Enrichment was quantified using Storm820 Phosphor Imager (Amersham Biosciences, Image Quant TL Software v.2002.01) as previously described^41^. Percentage enrichment was calculated using the value for the input sample to normalize.

### Statistical analysis

Data were analyzed using an unpaired Student's t-test when appropriate. A P-value of less than or equal to 0.05 was considered as statistically significant for observed changes. The standard error of the mean (SEM) is shown on graphs when appropriate.

## Supplementary Information

Supplementary Information.

## Data Availability

No datasets were generated or analyzed during the current study.
