# Supplementary material for: GATA4 blocks squamous epithelial cell gene expression in human esophageal squamous cells
Source: Sci Rep. 2021 Feb 5;11:3206. doi: 10.1038/s41598-021-82557-x (PMC7864948; doi:10.1038/s41598-021-82557-x)
Supplement: Supplementary file 1 — Supplementary Information. [file 41598_2021_82557_MOESM1_ESM.pdf]

GATA4 blocks squamous epithelial cell gene expression in human esophageal squamous cells

Roman Stavniichuk[1], Ann DeLaForest[1], Cayla A. Thompson[1], James Miller[2], Rhonda F. Souza[3], Michele A. Battle[1]*

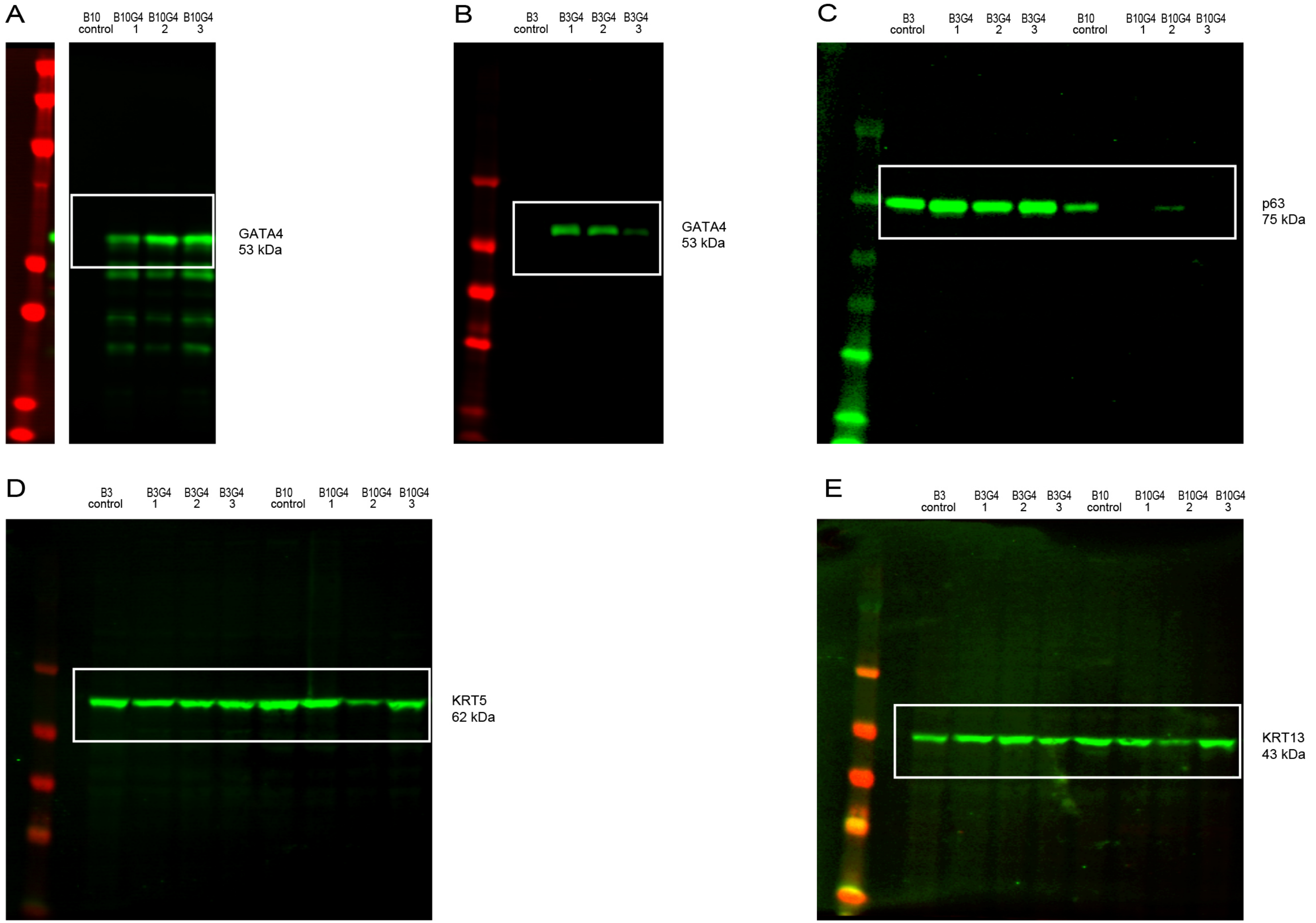

Supplementary Figure 1. Full immunoblots from which panels in Figures 1-3 were derived.
(A, B) Immunoblots corresponding to GATA4 panels in Figure 1. (A) shows NES-B10T cell clones. (B) shows NES-B3T cell clones.
(C-E) Immunoblots corresponding to p63, KRT5, and KRT13 panels Figures 2 and 3. NES-B3T clones are on the left of each blot and NES-B10T clones are on the right of each blot.
The molecular weight protein ladder is shown on the left in each blot.

A

p63 Bio-ChIP-PCR

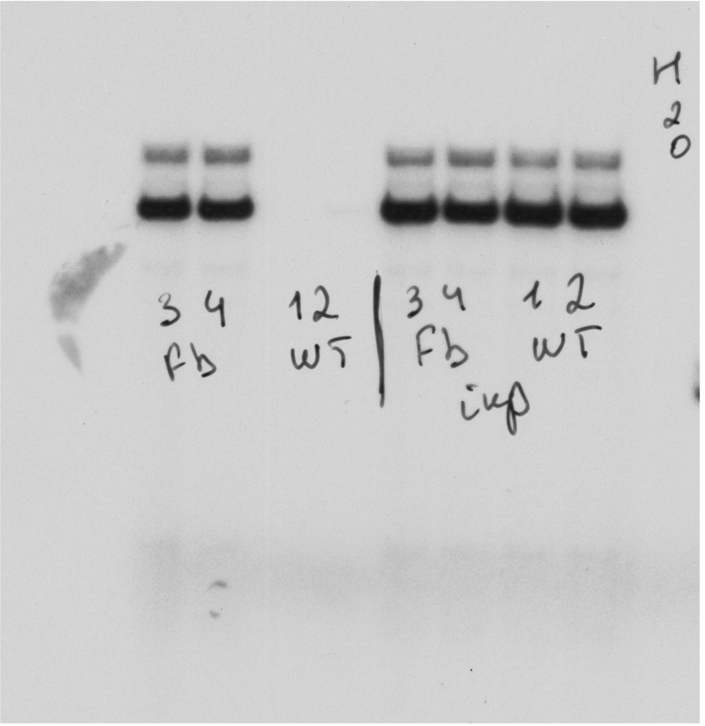

B

KRT5 Bio-ChIP-PCR

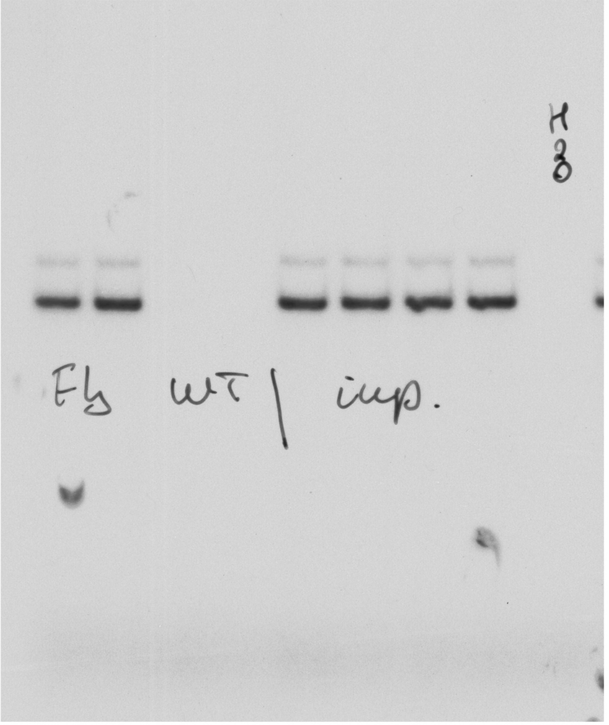

C

KRT15 Bio-ChIP-PCR

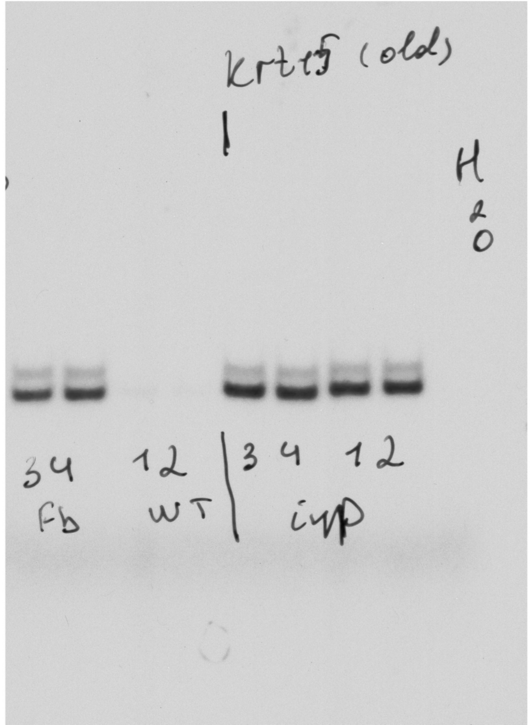

D

HPRT Bio-ChIP-PCR

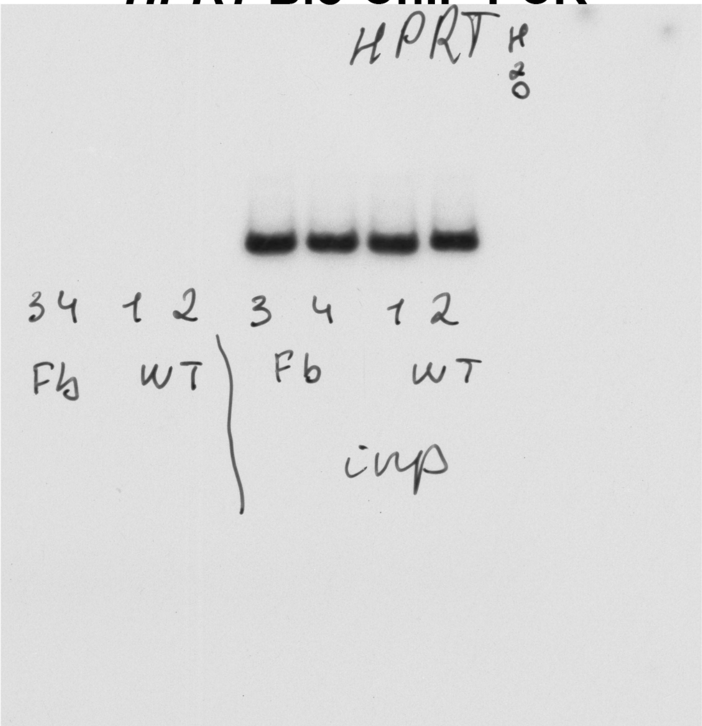

Supplementary Figure 2. Full autoradiographs from which panels in Figure 5 were derived. (A) shows Bio-ChIP-PCR for the *p63* gene promoter. (B) shows Bio-ChIP-PCR for the *KRT5* gene promoter. (C) shows Bio-ChIP-PCR for the *KRT15* gene promoter. (D) shows Bio-ChIP-PCR for the *HPRT*. Fb, GATA4-BIO lanes animals 3 and 4 labeled as GATA4-BIO1 and BIO2 in Figure 5; WT, GATA-WT lanes animals 1 and 2 labeled as GATA4-WT1 and WT2 in Figure 5; inp, input lanes; water, negative control zero DNA input PCR control labeled as no DNA in Figure 5.

A **GATA4** **B** **GAPDH**

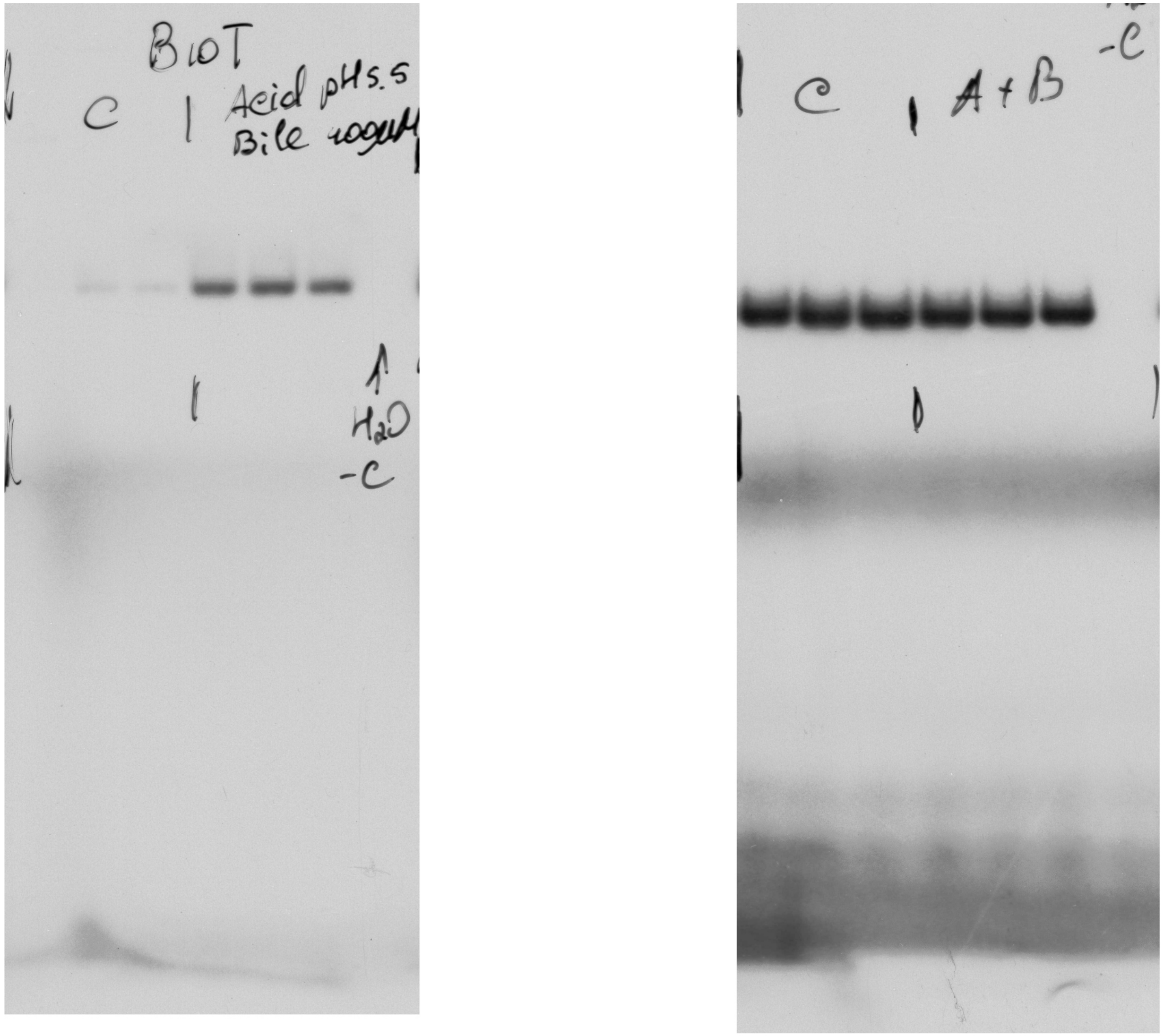

Supplementary Figure 3. Full autoradiographic film images used to construct Figure 7.
(A) GATA4 PCR; (B) GAPDH PCR. “C” refers to three technical replicates of non-treated NES-B10T cells labeled as Control in Figure 7.
“Acid Bile ” or “A+B” refer to three technical replicates of acid/bile treated NES-B10T cells labeled as Acid+Bile in Figure 7.
The right-most lane labeled “water”” or “-C” refers to a no DNA input negative control PCR reaction labeled as No DNA in Figure 7.
